# Fetal Meconium Peritonitis: A Clinical Study of Nine Cases

**DOI:** 10.1155/2022/8658999

**Published:** 2022-05-27

**Authors:** Fengping Fu, Xia Song, Fudan Huang, Hang Yuan, Li Xiao

**Affiliations:** Shandong Provincial Maternal and Child Health Care Hospital, Jinan City, Shandong Province, China

## Abstract

**Objective:**

To explore the prenatal ultrasonographic characteristics and pregnancy outcomes of fetal meconium peritonitis (FMP).

**Methods:**

Nine patients diagnosed with FMP by routine prenatal examination between January 2015 and December 2020 were identified. Both prenatal ultrasonographic characteristics and pregnancy outcomes associated with these patients were retrospectively analyzed.

**Results:**

The mean gestational age at the time of FMP diagnosis was 31.3 ± 4.8 weeks, and the mean gestational age of delivery was 35.1 ± 5.1 weeks. Prenatal ultrasonographic findings at the time of diagnosis in these patients included intestinal dilatation (9/9, 100%), intraperitoneal calcification (8/9, 88.9%), fetal ascites (5/9, 55.6%), intraperitoneal pseudocyst (5/9, 55.6%), and polyhydramnios (6/9, 66.7%). Analyses of the etiological basis for meconium peritonitis in 5 of the 8 live births that underwent surgical treatment revealed 4 cases of congenital volvulus and 1 case of jejunal atresia.

**Conclusion:**

The prenatal ultrasound manifestations of fetal meconium peritonitis are diverse, and the different grades of prenatal ultrasound manifestations can provide important information for the treatment of perinatal infants.

## 1. Introduction

FMP (fetal meconium peritonitis) is a rare form of sterile chemical peritonitis that can occur in utero, primarily as a consequence of the flow of meconium into the abdominal cavity due to the perforation of the fetal intestine [1]. The incidence of FMP is low, about 1 in 30,000 [2]. Owing to its rarity and the limited amount of research conducted on this condition to date, FMP was associated with a case fatality rate of up to 43.7%–59.6% in 2003 [3]. The neonatal mortality associated with this condition has, however, declined in recent years owing to new advances in prenatal diagnostics, with the live birth rate of affected neonates now ranging from 80%–92.3% [4–6]. Herein, we conducted a retrospective analysis of the ultrasonographic findings and perinatal outcomes associated with 9 cases of prenatally diagnosed FMP in an effort to provide a better basis for prenatal diagnosis and management of this potentially dangerous condition.

## 2. Materials and Methods

### 2.1. Subjects

In total, data from 9 patients diagnosed with FMP during routine ultrasonographic prenatal examinations from January 2015 to December 2020 were retrospectively analyzed. All pregnant women provided written informed consent to undergo these prenatal ultrasound examinations, and the ethics committee of our hospital approved the present retrospective study.

### 2.2. Research Methods

Clinical data pertaining to the 9 patients diagnosed with FMP were analyzed retrospectively, including prenatal ultrasound examination characteristics, the diagnosis made following postnatal imaging or surgical exploration, and associated neonatal outcomes. Correlations between prenatal ultrasonography and neonatal prognosis were additionally evaluated.

### 2.3. Diagnostic Criteria

Prenatal ultrasonographic findings of FMP are primarily associated with one or more of the following, with or without hyperhydramnios: (1) calcification in the abdominal cavity of the fetus; (2) fetal peritoneal effusion; (3) fetal intestinal dilatation; (4) pseudocyst in the fetal abdominal cavity [6]. The FMP ultrasonic classification standards are shown in Table 1 [7].

## 3. Results

### 3.1. General Data and Clinical Outcomes

The mean gestational age of the 9 patients in this study was 29.6 ± 5.3 years old, the mean gestational age at diagnosis was 31.3 ± 4.8 weeks, and the mean gestational age at delivery was 35.1 ± 5.1 weeks. Among the 9 patients, there were 8 live births and 1 case of induced labor. The mean birth weight of the resultant newborns (6 males, 3 females) was 2795.6 ± 908.5 g, and of these 9 patients, 7 underwent delivery via cesarean section (77.8%), fetal movement decreased in 3 cases (33.3%), and 2 exhibited amniotic fluid meconium contamination degree II or III (22.2%). Of these 9 patients, 2 had undergone prenatal screening for serum toxoplasma, rubella virus, cytomegalovirus, and herpes simplex virus, with no abnormal findings. Three cases underwent ultrasound-guided amniocentesis for prenatal diagnosis, with no findings consistent with pathogenicity, and one case underwent ultrasound-guided fetal abdominal puncture before birth.

For these 9 cases, prenatal ultrasound suggested potential meconium peritonitis with fetal intestinal obstruction or intestinal atresia. In total, 5 out of 8 live births underwent surgical treatment, leading to the confirmation of congenital volvulus in 4 cases and jejunal atresia in 1 case. The remaining 3 cases improved upon conservative treatment.

### 3.2. Prenatal Ultrasound Findings

Upon prenatal ultrasonographic assessment of these 9 cases, 8 (88.9%) exhibited visible fetal intraabdominal calcifications, 5 (55.6%) exhibited visible fetal ascites, 5 (5/9,55.6%) exhibited fetal intraabdominal pseudocyst, 6 (66.7%) exhibited hydramnios, and 1 exhibited fetal edema, fetal right heart enlargement, and pericardial effusion. The ultrasonographic characteristics and grades associated with these 9 cases are shown in Table 2.

## 4. Discussion

The pathogenesis of FMP is poorly understood but may be associated with congenital ileus [8], congenital intestinal wall dysplasia [9], intrauterine infection, or other factors, with some cases additionally being associated with cystic fibrosis [10], chromosomal malformations, or spontaneous intestinal perforation of unknown cause. Congenital intestinal obstruction is the most common cause of FMP [8] and may arise as a consequence of intestinal atresia, intussusception, or intestinal volvulus [8]. The clinical presentation of patients with FMP varies depending on the timing of fetal intestinal perforation and whether the perforation is closed. In this study, 4 cases of FMP were confirmed by surgery as being associated with congenital intestinal obstruction and intestinal perforation resulting from intestinal torsion, while 1 case was associated with congenital jejunal atresia. There are some reports suggesting that there may be cases of familial FMP [11], but further research on this topic is warranted.

The prenatal diagnosis of FMP is primarily dependent upon ultrasound-based screening and detection at present. Prenatal ultrasound scans can detect FMP at a median gestational age of approximately 32 weeks, with a median gestational age at delivery of about 36 weeks [4]. In the present report, we similarly observed an average gestational age at diagnosis of 31.3 ± 4.8 weeks and an average gestational age at delivery of 35.1 ± 5.1 weeks, in line with these prior reports. FMP can be associated with diverse ultrasonographic manifestations, with the most common such findings being fetal ascites, polyhydramnios, calcification in the fetal abdominal cavity, dilatation of the fetal intestinal loops, fetal edema, and pseudocyst formation [5]. The primary prenatal ultrasonography results used to guide the diagnosis of FMP include ascites, abdominal calcification, and intestinal perforation and/or dilatation. In the present study, the most common ultrasonographic findings were fetal intestinal dilatation, followed by fetal intraperitoneal calcification, fetal ascites with intestinal perforation and dilation of the oop intestine, and 1 case presenting with hyperhyniotic amniotic fluid. Some researchers classify FMP into three types depending on the presence of specific features, with type I FMP presenting with ascites, type II FMP being associated with cysts as a consequence of local ascites accumulation and adhesion in the bowel and greater omentum having resulted in pseudocyst formation, and type III FMP being associated with fibrous adhesions as a consequence of calcium salt closed perforation caused by the sedimentation of the bowel perforation position [4, 5, 12]. With intraperitoneal calcification as the common phenomenon, Zangheri et al. divided FMP into four levels according to the presence of other abnormal ultrasonographic situations: level 0 refers to the cases where only intraperitoneal calcification is observed, level 1 refers to the cases where intraperitoneal calcification and one abnormal ultrasonographic situation are observed, level 2 refers to the cases where intraperitoneal calcification and two abnormal ultrasonographic situations are observed, and level 3 refers to the cases where intraperitoneal calcification and three abnormal ultrasonographic situations are observed [7]. The majority of cases in the present study were of grade 2 or 3. In some prior studies, magnetic resonance imaging (MRI) has been used to guide the prenatal diagnosis of FMP, but at present prenatal ultrasound remains the primary diagnostic approach for this condition.

Prenatal diagnostic tests such as fetal chromosomal karyotyping, gene microarrays, and TORCH screening are recommended in cases of suspected prenatal FMP. If a gene fragment suspected to be pathogenic is identified, patients should undergo antenatal consultation to determine whether to continue the pregnancy. In this study, two patients underwent prenatal diagnosis, and no suspected pathogenic gene fragments were identified.

In this study, 7 of the 9 patients delivered via cesarean section, but there is no evidence that cesarean delivery can improve neonatal outcomes associated with FMP. The postnatal treatment and prognosis of FMP are closely linked to whether or not there is evidence of intestinal perforation and intestinal obstruction. According to the grading system proposed by Zangheri et al., grade 0 fetuses require no surgical intervention after birth, whereas approximately 50% of grade 1 fetuses will require surgical treatment, and almost all grade 2 and 3 fetuses will require postnatal surgery [7]. In this study, 5 of the 8 live neonates underwent postnatal surgery, all of whom were Zangheri grade 2 or grade 3 cases. Some studies have shown that pseudocyst, intestinal loop dilatation, and ascites are predictors of the need for neonatal surgical treatment [13]. Early diagnosis and early treatment are of particular importance in neonates with FMP complicated by intestinal perforation and obstruction. The prognosis of all children in the present study was good.

In summary, while FMP remains a rare condition, its timely diagnosis and postnatal management are important as a means of improving neonatal prognosis. Prenatal ultrasonography is a key means of diagnosing FMP, which can allow for the dynamic monitoring of disease progression during pregnancy while offering guidance regarding neonatal clinical outcomes.

## Figures and Tables

**Table 1:** Zangheri's FMP grading system [7].

Classification		Sonographic findings
0		Simple intraperitoneal calcification
1	1A	Intraperitoneal calcification with ascites
	1B	Intraperitoneal calcification with pseudocyst
	1C	Intraperitoneal calcification with intestinal loop dilatation
2		Intraperitoneal calcification with the above two kinds of ultrasound abnormalities
3		Intraperitoneal calcification with ≥3 of the above related ultrasonographic abnormalities

**Table 2:** Ultrasonic characteristics and classifications.

Serial number	Sonographic findings	Classification
1	Fetal intestinal dilatation, fetal intraperitoneal calcification, fetal ascites, fetal intraperitoneal pseudocyst, hydramnios	3
2	Fetal intestinal dilatation, fetal peritoneal calcification, fetal ascites	2
3	Fetal intestinal dilatation, fetal peritoneal calcification, fetal ascites	2
4	Fetal intestinal dilatation, fetal intraperitoneal pseudocyst, hydramnios	Unrated
5	Fetal intestinal dilatation, fetal peritoneal calcification, fetal ascites, excessive amniotic fluid	2
6	Fetal intestinal dilatation, fetal peritoneal calcification, hydramnios	1C
7	Fetal intestinal dilatation, fetal peritoneal calcification, fetal peritoneal pseudocyst	2
8	Fetal intestinal dilatation, fetal intraperitoneal calcification, fetal ascites, fetal intraperitoneal pseudocyst, fetal hyperhydramnios, fetal edema, fetal right heart dilatation with pericardial effusion	3
9	Fetal intestinal dilatation, fetal peritoneal calcification, fetal intraperitoneal pseudocyst, hydramnios	2

## Data Availability

The datasets used to support the findings of this study are available from the corresponding author on reasonable request.
